# Compositional patterns of device-measured movement behaviour in juvenile idiopathic arthritis: results from the multicentre ActiMON study

**DOI:** 10.1186/s13075-025-03714-5

**Published:** 2025-12-17

**Authors:** Florian Milatz, Lisa Voigt, Jens Klotsche, Samuel Tomczyk, Tilmann Kallinich, Ralf Trauzeddel, Daniel Windschall, Sandra Hansmann, Nadja Baumeister, Johannes-Peter Haas, Moritz Klaas, Hermann Girschick, Joachim Peitz-Kornbrust, Peter Böhm, Julius Wiegand, Stefan N. Willich, Alexander Burchartz, Kirsten Minden

**Affiliations:** 1 Programme area Epidemiology and Health Services Research, German Rheumatology Research Center Berlin, a Leibniz-Institute, Berlin, Germany; 2 Institute for Social Medicine, Epidemiology and Health Economics, Charité - Universitätsmedizin Berlin, corporate member of Freie Universität Berlin and Humboldt- Universität zu Berlin, Berlin, Germany; 3 Partner site Berlin, German Center for Child and Adolescent Health (DZKJ), Berlin, Germany; 4 IGES Institute, Berlin, Germany; 5 Institute of Medical Psychology and Medical Sociology, University of Rostock, Rostock, Germany; 6 Partner site Greifswald/Rostock, German Center for Child and Adolescent Health (DZKJ), Rostock, Germany; 7 Department of Paediatric Respiratory Medicine, Immunology and Critical Care Medicine, Charité – Universitätsmedizin Berlin, corporate member of Freie Universität Berlin and Humboldt- Universität zu Berlin , Berlin, Germany; 8 Systems Rheumatology, German Rheumatology Research Center Berlin, a Leibniz-Institute, Berlin, Germany; 9 Department of Paediatrics, Helios Klinikum Berlin-Buch, Berlin, Germany; 10 Clinic of Paediatric and Adolescent Rheumatology, Northwest German Centre for Rheumatology, St. Josef-Stift Sendenhorst, Sendenhorst, Germany; 11 Medizinische Fakultät, Universität Halle-Wittenberg, Halle, Germany; 12 Department of Neuropediatrics Developmental Neurology and Social Paediatrics, University of Tuebingen, Tuebingen, Germany; 13 Department Health and Sport Sciences, Technical University of Munich, Munich, Germany; 14 German Centre for Pediatric and Adolescent Rheumatology, Garmisch-Partenkirchen, Germany; 15 Children’s Hospital, Vivantes Klinikum Friedrichshain, Berlin, Germany; 16 Department of Pediatrics, Children’s Hospital Sankt Augustin, Sankt Augustin, Germany; 17 Deutsche Rheuma-Liga e. V., Bonn, Germany; 18 Institute for Sports and Sports Science, Karlsruhe Institute of Technology, Karlsruhe, Germany

**Keywords:** Juvenile idiopathic arthritis, Physical activity, Sedentary behaviour, Movement behaviour, Accelerometry, Children and adolescents, Compositional data analysis

## Abstract

**Background:**

Children and adolescents with juvenile idiopathic arthritis (JIA) are at increased risk for long-term physical and psychosocial complications, making physical activity (PA) and sedentary behaviour (SB) key modifiable lifestyle factors. Although increasingly acknowledged as relevant, data on the daily distribution of these behaviours in JIA remain scarce. This study aimed to (1) describe the time-use composition of SB and PA intensities in young people with JIA, (2) identify correlates of greater relative time spent in SB, and (3) compare movement behaviour patterns to matched population controls using a compositional data analysis (CoDA) approach.

**Methods:**

Patients aged 10–20 years with JIA and individually matched population controls wore hip-worn accelerometers (ActiGraph wGT3X-BT) for eight consecutive days. Movement behaviours were categorized into SB, light-intensity PA, and moderate-to-vigorous PA (MVPA) using validated, age-specific cut-points. CoDA with log-ratio transformations was used to model associations and compare groups. Diifferences in movement composition were assessed using adjusted multivariate analysis of variance (MANOVA).

**Results:**

Data from 126 matched pairs (mean age: 15.0 ± 2.1 years; 67% female) were analysed. Patients spent, on average, 86% of waking time in SB, 8% in light-intensity PA, and 6% in MVPA. Overall, 76% did not meet the WHO recommendation of an average of ≥ 60 min of MVPA per day. Among those who did, 87% still spent ≥ 75% of their wear time sedentary. A greater proportion of SB relative to PA was associated with female gender (B = 0.13; *p* = 0.042), higher age (B = 0.06; *p* < 0.001), and higher BMI (B = 0.01; *p* = 0.049). Compared to controls, patients spent more time in SB, less in light-intensity PA, and slightly more in vigorous PA (all *p* < 0.001). Group differences remained significant after adjustment and were consistent across weekdays and weekends.

**Conclusions:**

Young people with well-controlled JIA show a distinctly unbalanced movement behaviour composition compared to controls, marked by a predominance of sedentary behaviour—even among those meeting PA guidelines. This highlights the limitations of threshold-based definitions and underscores the importance of assessing full daily movement patterns. Promoting light-intensity PA may offer a feasible strategy to reduce sedentary time, particularly in girls, older adolescents, and individuals with higher BMI.

**Trial registration:**

The study was registered in the German Clinical Trials Register (DRKS00022258).

**Supplementary Information:**

The online version contains supplementary material available at 10.1186/s13075-025-03714-5.

## Background

Juvenile idiopathic arthritis (JIA) encompasses a clinically diverse group of rheumatic inflammatory conditions of unknown etiology. It can present with systemic signs, isolated arthritis, or extra-articular inflammations such as psoriasis and uveitis. With a prevalence of 16–150 per 100,000 children in Western countries, JIA ranks among the most common chronic diseases in the pediatric population [1]. Although some patients enter spontaneous permanent remission, up to 78% of patients require ongoing or recurrent treatment in adulthood [2].

Emerging evidence indicates that individuals with JIA are at increased risk for both physical and mental comorbidities compared to the general population [3–5], underlining the importance of early preventive strategies to mitigate long-term health consequences.

In this context, physical activity (PA) and sedentary behaviour (SB) have gained attention as key modifiable lifestyle factors influencing physical and mental health outcomes across populations [6]. PA refers to any bodily movement produced by skeletal muscles that requires energy expenditure, whereas SB describes activities such as sitting, reclining, or lying down that involve minimal energy expenditure (≤ 1.5 Metabolic equivalents) [6]. Amongst other eminent scientific authorities [7, 8], the World Health Organization (WHO) recommends that children and adolescents accumulate at least 60 min of moderate-to-vigorous PA (MVPA) on average per day and minimize sedentary time [6].

While such guidelines traditionally address PA and SB as independent targets, time spent in one behaviour inevitably reduces time in others. This time-bound interdependence has prompted a shift in perspective, away from isolated analyses of single behaviours toward an integrated, compositional understanding of daily movement patterns [9]. Rather than adjusting only partially for confounding behaviours, recent work emphasizes the importance of appropriately modelling the full time-use composition [10, 11].

Despite the clinical relevance, evidence of device-based movement behaviour patterns in children and adolescents with JIA remains scarce. Existing studies are limited by small or selected samples, suboptimal device placement, or lack of SB data. Moreover, they did not use age-validated cut-points, compositional approaches, or standardized data processing aligned with reference protocols [12–19].

To address these limitations, the present study is the first to examine movement behaviour composition in JIA using compositional data analysis (CoDA). Drawing on one of the largest, first-time multicenter accelerometer datasets in this population, this study aimed to:

1. Describe device-measured time-use compositions of SB, light PA, moderate PA, and vigorous PA stratified by sociodemographic and clinical characteristics;
2. Identify sociodemographic and disease-related correlates of greater relative time spent in SB;
3. Compare movement behaviour compositions of JIA patients to individually matched population controls.

To ensure methodological rigor and future comparability, all analyses were conducted on data fulfilling the inclusion criteria of the International Children’s Accelerometry Database (ICAD) [20].

## Methods

### Study design

The cross-sectional ActiMON study (Activity monitoring in adolescents and young adults with inflammatory rheumatic musculoskeletal diseases) is a multicenter study investigating movement patterns and their determinants in young people with rheumatic musculoskeletal diseases. The study was part of the TARISMA research network (Targeted Risk Management in Musculoskeletal Diseases), a multi-site prospective study aiming to improve healthcare among patients with musculoskeletal diseases in Germany. Below we describe the methods relevant to a sub-study within ActiMON. The study was registered in the German Clinical Trials Register (DRKS00022258). Ethical approval was obtained from the ethics committee of Charité – Universitätsmedizin Berlin and all participating regional ethics boards. Further details are provided in the “Declarations” section.

### Patients

Inclusion criteria for patients were: (1) age between 10 and 20 years at documentation, (2) diagnosis of JIA according to the International League of Associations for Rheumatology (ILAR) criteria [21], (3) disease duration of at least 6 months, and (4) physician global assessment of disease activity < 4 (NRS 0–10; 0 = lowest, 10 = highest). Exclusion criteria included: (1) recent surgery (within the past 3 months) limiting PA, (2) comorbid neurological, cardiac, or pulmonary conditions restricting PA, (3) high-dose steroid therapy (> 0.2 mg/kg or > 10 mg/day) in the past 4 weeks, (4) intra-articular steroid injections within 4 weeks, (5) pregnancy or breastfeeding, and (6) language or cognitive barriers to participation.

Eligible patients were recruited from seven paediatric rheumatology centers across Germany (northwest, south, northeast) during routine care visits between June 2021 and June 2023. Recruitment continued until the targeted sample size was reached. Written informed consent was obtained from all participants and/or legal guardians following verbal and written information about the study content.

### Controls

Individually age- and gender-matched controls from the German general population were drawn from the Motorik-Modul study (MoMo), which is part of the nationwide German Health Interview and Examination Survey for Children and Adolescents (KiGGS) [22]. MoMo participants were recruited using a stratified multistage sampling strategy across 167 locations [23]. For the present analysis, control data were selected from a period that overlapped with the COVID-19 pandemic to ensure contextual comparability with the patient sample. MoMo was designed to monitor PA and fitness levels in children and adolescents and to explore related health outcomes. Detailed study protocols for KiGGS and MoMo are available elsewhere [24].

### Physical activity and sedentary behaviour

PA and SB were obtained using tri-axial ActiGraph Model wGT3X-BT accelerometers (ActiGraph LLC, Pensacola, FL). Each accelerometer was initialized using ActiLife Lite version 6.13.5 (ActiGraph LLC, Pensacola, FL) following a standardized protocol. Devices were then handed out to patients. As participants received their device at different times throughout their examination day, each accelerometer was set up to start recording the day after routine consultation (at 12:00 AM).

Since wearing an accelerometer can influence individuals’ behaviour (known as measurement reactivity [25]), two approaches were taken: First, each participant received standardized instructions that only contained as much information as necessary about the outcome measure (e.g., explaining “measuring activity” rather than “measuring steps”). Relevant information was given verbally by trained clinical staff and visualised by watching a short project film (https://youtu.be/lnWelV8hm60). Key aspects (e.g. placement, wearing times and returning the device) were summarized in an information sheet to take home. Second, recordings from the first measurement day, intended as a familiarization phase, were excluded from further data processing and analyses.

Participants wore the device laterally on the right hip (top of right anterior superior iliac spine) attached to an elastic belt. To ensure inclusion of weekdays and weekend days as recommended [26, 27], participants were instructed to wear the accelerometer on eight consecutive days during waking hours and to put it off for water-based activities such as morning hygiene or swimming. Recording ended automatically at midnight on the eighth day.

To avoid conflating sedentary time with night-time sleep in cases the accelerometer was not removed, participants kept a non-wear log. Once the recording was complete, log and accelerometer were returned to the clinic by postal mail in a prepaid envelope. Data were collected exclusively during school/work weeks (excluding holidays) to ensure standardized daily routines.

Raw data were aggregated into 1-second epochs as recommended for children and adolescents [28, 29] and processed with ActiLife full version 6.13.5. Activity intensities were categorized using validated vertical axis cut-points by Romanzini et al. [30], originally defined for 15-second epochs and proportionally adjusted to match the epoch length applied in our analysis. Participants spending ≥ 75% of total wear time in SB were classified as predominantly sedentary, in line with definitions by the Sedentary Behaviour Research Network (SBRN) [31].

Accelerometer datasets were considered valid based on ICAD standards [20], requiring ≥ 8 h of wear time on ≥ 4 weekdays and ≥ 1 weekend day. All data processing procedures followed the protocols used in the MoMo study [32] and recommendations by Migueles et al. [29] (see Table S1).

As data collection took place during the post-COVID-19 adjustment period, participants were asked to self-report whether their current movement behaviour had changed compared to the pre-pandemic period. Response options were “less physically active”, “about the same,” and “more physically active”.

### Demographic and clinical data

Sociodemographic and clinical data were collected at the time of accelerometer distribution, using standardized physician- and patient-reported questionnaires from the National Paediatric Rheumatologic Database (NPRD), a well-established registry for paediatric patients in Germany. Detailed descriptions of the NPRD have been published by Minden et al. [33] and Klotsche et al. [34].

Paediatric rheumatologists reported demographic and clinical information including birth month/year, gender, diagnosis, age at disease onset, height, and weight. BMI was calculated and categorized using age- and gender-specific percentiles from German reference data [35], which are standard in national epidemiologic studies. Categories included underweight (< 10th percentile), normal weight (10th–90th), overweight (> 90th), and obesity (> 97th).

Disease activity was assessed using the physician’s global assessment (PGA) on a numerical rating scale (NRS) ranging from 0 (no disease activity) to 10 (very severe disease activity). Rheumatologists also documented the number of joints with active arthritis and current pharmacological treatment, including non-steroidal anti-inflammatory drugs (NSAIDs), glucocorticoids (GCs), vitamin D supplements, conventional synthetic disease-modifying antirheumatic drugs (csDMARDs), and biologic DMARDs (bDMARDs).

Patients reported on functional ability using the German version of the Childhood Health Assessment Questionnaire (C-HAQ) [36]. The resulting disability index ranges from 0 to 3, with a score of 0 indicating no functional impairment and higher scores reflecting mild, moderate, or severe disability. Additional self-reports captured overall well-being, pain, fatigue, perceived disease activity, and coping (all via NRS 0–10). Patients were also asked whether they had one or more long-term health conditions in addition to their rheumatic disease that had been diagnosed by a physician (yes/no). If answered ‘yes’, they could select from a predefined list of common conditions and/or specify other diagnoses in a free-text field.

The county of their residence was used to assign patients to spatial planning regions and consequently to the German Index of Socioeconomic Deprivation (GISD) [37]. The GISD is open to be used for research at the data repository of the German GESIS Leibniz-Institute for the Social Sciences (10.7802/1460). The GISD encompasses regional data on education, occupation, and income, the three dimensions of the socioeconomic status as it is usually defined in social epidemiology. The methodology used to develop this index has been described in detail previously [38]. To harmonize the analyses, deprivation was divided into terciles: low, medium and high.

Based on physician- and patient-reported data, disease activity was assessed using the clinical Juvenile Arthritis Disease Activity Score in 10 joints (cJADAS-10) [39], which combines active joint count, PGA, and patient-reported well-being.

### Statistical analyses

To minimize potential reactivity bias from wearing the accelerometer, recordings from the first day were excluded from all analyses [40]. Descriptive statistics for categorical variables are presented as absolute and relative frequencies, while continuous variables are reported as means and standard deviations.

For aim 1, we described the time-use composition of SB, light-intensity PA, and MVPA as a percentage of total wear time, stratified by sociodemographic and clinical characteristics. The proportion of participants meeting the WHO recommendation of an average of ≥ 60 min of MVPA per day was also calculated.

For aim 2, we used multiple linear regression models to examine correlates of SB relative to light-intensity PA and MVPA. Movement behaviours were expressed using isometric log-ratio (ILR) transformation, with the first pivot coordinate representing the log-ratio of SB to the geometric mean of the remaining behaviours [9, 10, 41]. ILR-transformed values were regressed on sociodemographic and clinical predictors, adjusting for season of data collection. Results are reported as both unstandardized and standardized regression coefficients with 95% confidence intervals.

For aim 3, we compared the overall movement behaviour composition between JIA patients and individually age- and gender-matched population controls. Wilcoxon signed-rank tests and McNemar tests were used for matched comparisons of continuous and categorical variables, respectively. Multivariate analysis of variance (MANOVA) based on ILR transformation was used to compare full compositions. ILR allows for a symmetrical, unconstrained analysis of all components [9]. Partial eta squared (η^2^) was used as a measure of effect size and the corresponding p-value as a metric for evaluating statistical significance. Models were adjusted for BMI, socioeconomic deprivation, and season. Age and gender were not included as covariates due to matching. Analyses were performed for all days combined, and separately for weekdays and weekend days.

To assess potential selection bias, sociodemographic and clinical characteristics of participants with and without valid accelerometer data were compared using Welch’s test. Additionally, to examine potential temporal effects across the recruitment period, a sensitivity analysis was performed comparing movement behaviour compositions between participants recruited earlier (2021–mid 2022) and those recruited later (mid 2022–2023).

All compositional data transformations were performed in Stata version 18.5 (StataCorp), where accelerometer data were also cleaned and prepared for analysis. Regression modelling was conducted in IBM SPSS Statistics version 26.0 (IBM Corp., Armonk, NY, USA). Visualisations and graphics were generated in R version 4.3.2 using the packages robCompositions, zCompositions, ggplot2, and ggtern. The compositional data analytical strategy followed best-practice guidance in time-use epidemiology [9, 41]. A p-value < 0.05 was considered statistically significant. Details on wear-time validation and data transformation are provided in Supplementary Table S1.

## Results

### Participants

In total, 139 JIA patients participated in the study, of whom 126 (91%) contributed valid accelerometer data to the analyses. Participants’ mean age was 15.0 years (SD = 2.1) and 67% (*n* = 84) were female. About two thirds of the patients were classified as having normal weight and a similar proportion resided in medium deprived regions. 28.2% of patients reported at least one comorbidity in addition to JIA. The most frequently reported conditions were allergies (47.1%), asthma (20.6%), and uveitis, mental health disorders, or ADHD (each 11.8%).

Comparisons between patients with valid and invalid data showed no significant differences in basic characteristics. Further details on patients’ sociodemographic and clinical characteristics are shown in Table 1.

**Table 1:** Characteristics of JIA patients, stratified by age group

Variables	Total (*n* = 126)	Age group 10 to < 14 years (*n* = 46)	Age group 14 to < 16 years (*n* = 42)	Age group 16 to 20 years (*n* = 38)
Sociodemographic/anthropometric data				
Age, years, mean ± SD	15.0 ± 2.1	12.7 ± 0.9	15.2 ± 0.6	17.5 ± 0.9
Female, no. (%)	84 (66.7)	30 (65.2)	27 (64.3)	27 (71.1)
BMI, mean ± SD	21.1 ± 4.1	19.8 ± 3.5	21.4 ± 4.8	22.3 ± 3.5
Underweight, no. (%)	13 (10.6)	3 (6.7)	7 (17.5)	3 (7.9)
Normal weight, no. (%)	84 (68.3)	32 (71.1)	23 (57.5)	29 (76.3)
Overweight/obesity, no. (%)	26 (21.1)	10 (23.3)	10 (25.0)	6 (15.8)
JIA specific data				
Disease duration, years, mean ± SD	8.3 ± 4.5	7.0 ± 3.8	8.6 ± 4.7	9.4 ± 4.7
Age at disease onset, years, mean ± SD	6.7 ± 4.4	5.7 ± 3.6	6.5 ± 4.7	8.1 ± 4.7
JIA category, no. (%)				
RF-positive polyarthritis	7 (5.6)	2 (4.3)	0 (0.0)	5 (13.2)
RF-negative polyarthritis	31 (24.6)	15 (32.6)	9 (21.4)	7 (18.4)
Systemic JIA	3 (2.4)	1 (2.2)	1 (2.4)	1 (2.6)
Persistent oligoarthritis	38 (30.2)	16 (34.8)	14 (33.3)	8 (21.1)
Extended oligoarthritis	20 (15.9)	5 (10.9)	9 (21.4)	6 (15.8)
Psoriatic arthritis	7 (5.6)	1 (2.2)	2 (4.8)	4 (10.5)
Enthesitis-related arthritis	14 (11.1)	5 (10.9)	5 (11.9)	4 (10.5)
Unclassified JIA	6 (4.8)	1 (2.2)	2 (4.8)	3 (7.9)
cJADAS-10, 0–30, mean ± SD	2.4 ± 2.9	1.8 ± 2.3	3.1 ± 3.6	2.5 ± 2.9
PGA score, NRS 0–10, mean ± SD	0.5 ± 0.9	0.5 ± 0.8	0.7 ± 1.1	0.4 ± 0.6
Inactive disease*, no (%)	72 (61.0)	27 (62.8)	25 (59.5)	20 (60.6)
No. of joints with active disease, mean ± SD	0.4 ± 1.3	0.3 ± 0.9	0.7 ± 1.8	0.3 ± 0.7
Current drug therapy, no. (%)				
NSAIDs	16 (13.9)	6 (14.6)	4 (9.8)	6 (18.2)
Systemic GCs	0 (0.0)	0 (0.0)	0 (0.0)	0 (0.0)
Vitamin D	13 (11.3)	6 (14.6)	4 (9.8)	3 (9.1)
Any biologic DMARD	61 (51.7)	22 (50.0)	20 (48.8)	19 (57.6)
Any conventional synthetic DMARD	50 (42.4)	19 (43.2)	20 (48.8)	11 (33.3)
Patient-reported data, mean ± SD				
C-HAQ total score (0–3)	0.14 ± 0.3	0.11 ± 0.2	0.14 ± 0.3	0.19 ± 0.3
Overall well-being (NRS 0–10)	1.6 ± 1.9	1.1 ± 1.4	1.7 ± 2.1	2.0 ± 2.2
Pain intensity (NRS 0–10)	1.5 ± 2.0	1.0 ± 1.4	1.4 ± 2.1	2.0 ± 2.4
Fatigue (NRS 0–10)	1.7 ± 2.5	1.0 ± 1.5	1.4 ± 2.4	2.8 ± 3.2
Coping (NRS 0–10)	0.9 ± 1.5	0.6 ± 1.0	0.9 ± 1.7	1.2 ± 1.7
Disease activity (NRS 0–10)	1.1 ± 1.8	0.8 ± 1.5	1.6 ± 2.3	1.1 ± 1.7
Any comorbidity, no. (%)	35 (28.2)	10 (21.7)	11 (26.8)	14 (37.8)

*JIA,* juvenile idiopathic arthritis; *RF,* rheumatoid factor; *cJADAS-10,* 10-joint clinical Juvenile Arthritis Disease Activity Score; *PGA,* physician’s global assessment; *C-HAQ,* Childhood Health Assessment Questionnaire; *GC,* glucocorticoid; *DMARD,* disease-modifying antirheumatic drug; *NRS 0–10, * Numerical Rating Scale (0 = best, 10 = worst)

*Defined by a PGA score of zero

### Physical activity and sedentary behaviour in JIA patients

On average, patients wore their accelerometer 14.3 h per day (SD = 2.0) across 6.6 valid days (SD = 0.6). Daily wear time was distributed as follows: 12.3 h in SB, 1.2 h in light PA, 0.2 h in moderate PA, and 0.6 h in vigorous PA (Table 2). Overall, 76% of patients failed to meet the WHO recommendation of an average of at least 60 min of MVPA per day. Even among those meeting the WHO MVPA guideline, 87% were still classified as predominantly sedentary, defined as spending ≥ 75% of their total wear time in SB.

**Table 2:** Physical activity and sedentary behaviour in children and adolescents with JIA, stratified by sociodemographic and clinical variables

	*N*	SB (percentage day^− 1^) Mean ± SD	LPA (percentage day^− 1^) Mean ± SD	MVPA (percentage day^− 1^) Mean ± SD	Meets WHO PA Rec^6^ *N* (%)
Total	126	85.8 ± 4.7	8.4 ± 3.1	5.8 ± 2.4	30 (23.8)
Gender					
Female	84	86.4 ± 4.3	7.9 ± 2.8	5.6 ± 2.2	18 (21.4)
Male	42	84.5 ± 5.4	9.3 ± 3.5	6.2 ± 2.7	12 (28.6)
Iso-BMI category ^a^					
Underweight	13	87.3 ± 3.0	7.4 ± 1.5	5.2 ± 2.2	2 (15.4)
Normal weight	84	85.7 ± 4.6	8.2 ± 2.7	6.0 ± 2.5	23 (27.4)
Overweight/obesity	26	85.6 ± 5.2	9.0 ± 3.9	5.4 ± 2.0	4 (15.4)
Age group (years)					
10–13	46	83.6 ± 4.8	10.3 ± 3.0	6.0 ± 2.5	11 (23.9)
14–16	42	86.7 ± 4.5	7.6 ± 2.8	5.7 ± 2.1	10 (23.8)
17–20	38	87.5 ± 4.1	6.9 ± 2.2	5.6 ± 2.7	9 (23.7)
Socioeconomic deprivation^b^					
lowest	22	86.5 + 4.0	7.8 + 2.7	5.6 + 2.2	4 (18.2)
Medium low to high	79	85.1 + 5.1	8.9 + 3.3	6.0 + 2.5	23 (29.1)
highest	17	86.4 + 3.8	7.7 + 2.7	5.8 + 2.1	3 (17.6)
Disease duration (years)					
0–5	43	84.6 ± 4.7	9.2 ± 3.5	6.2 ± 2.2	13 (30.2)
6–10	44	85.5 ± 4.8	8.7 ± 2.7	5.8 ± 2.7	11 (25.0)
11–16	38	87.5 ± 4.3	7.2 ± 2.7	5.3 ± 2.2	6 (15.8)
Age at disease onset (years)^c^					
0–3	40	86.7 ± 4.4	7.7 ± 2.7	5.5 ± 2.3	9 (22.5)
4–8	38	85.0 ± 5.3	8.9 ± 3.1	6.1 ± 2.7	10 (26.3)
9–16	46	85.6 ± 4.6	8.6 ± 3.3	5.8 ± 2.2	11 (23.9)
JIA category					
RF-positive polyarthritis	7	88.4 ± 3.0	7.1 ± 1.5	4.5 ± 1.5	1 (14.3)
RF-negative polyarthritis	31	85.6 ± 5.8	8.7 ± 3.5	5.7 ± 2.8	6 (19.4)
Systemic JIA	3	87.0 ± 2.7	8.2 ± 1.9	4.8 ± 0.8	0 (0)
Persistent oligoarthritis	38	85.1 ± 4.8	8.9 ± 3.4	6.0 ± 2.1	12 (31.6)
Extended oligoarthritis	20	86.4 ± 3.8	7.6 ± 2.4	5.9 ± 2.2	3 (15.0)
Psoriatic arthritis	7	89.0 ± 3.5	6.4 ± 2.2	4.5 ± 1.9	1 (14.3)
ERA	14	84.2 ± 3.9	9.2 ± 2.9	6.6 ± 2.7	4 (28.6)
Unclassified JIA	6	85.6 ± 5.4	8.4 ± 2.7	6.0 ± 3.1	3 (50)
cJADAS-10 (0–30)					
< 1	47	86.2 ± 4.6	8.1 ± 2.6	5.7 ± 2.6	9 (19.1)
1–2.5.5	37	85.2 ± 4.6	9.0 ± 3.3	5.8 ± 1.9	10 (27.8)
> 2.5	42	86.0 ± 5.1	8.2 ± 3.4	5.8 ± 2.5	10 (23.8)
C-HAQ total score (0–3)^d^					
0	85	85.9 ± 4.8	8.4 ± 3.1	5.7 ± 2.4	17 (20.0)
> 0	37	85.6 ± 4.8	8.5 ± 3.1	5.9 ± 2.4	12 (32.4)

*JIA,* juvenile idiopathic arthritis;* ERA,* Enthesitis-related arthritis, *WHO,* World Health Organization; *PA,* physical activity, *%,* Percentage; *Rec,* Recommendations of ≥ 60 min MVPA on average per day; *SB,* sedentary behaviour; *LPA,* light intensity PA, *MVPA,* moderate-to-vigorous intensity PA

^a^Missing information on BMI in 1 patient

^b^Missing information on socioeconomic deprivation in 8 patients

^c^Missing information on age at disease onset in 2 patients

^d^Missing information on C-HAQ in 4 patients

Descriptive analyses showed that 14 to 16-year-olds (8%) and 17 to 20-year-olds (7%) spent less time in light-intensity PA than 10 to 13-year-olds (10%) and more time in SB (87% and 88% vs. 84%). No relevant age group differences were found in the proportion of patients reaching the WHO recommendation (Table 2).

On average, females spent more time in SB than males (86% compared to 84%) and achieved the WHO recommended minimum level of PA proportionately less often (21% compared to 29%). Based on descriptive statistics (univariate), the proportion of sufficiently physically active patients decreased with increasing disease duration. As shown in detail in Table 2, slight differences in movement behaviour composition were found between JIA categories. Patients with polyarthritis (18%) achieved the WHO recommendations proportionately less often than patients with oligoarthritis (25%). With regard to disease activity (cJADAS-10) and functional disability (C-HAQ), no relevant differences in movement behaviour composition were observed (Table 2). Overall, time-use composition of movement behaviours did not differ between weekdays and weekend days.

Additionally, 70% of patients self-reported no change in their overall movement behaviour compared to pre-pandemic times, while 14% reported being less and 16% more physically active. A sensitivity analysis comparing early (2021–mid 2022) and later (mid 2022–2023) recruited participants revealed no significant differences in movement behaviour composition.

### Factors associated with patients’ relative sedentary time

Compositional regression analysis showed that greater relative time in SB (compared to PA components) was associated with female gender, older age, and higher BMI, adjusted for season of data collection (Table 3).

**Table 3:** Factors associated with sedentary behaviour in patients with JIA (*n* = 126)

Dependent variable: Amount of time spent in sedentary behaviour relative to total wear time and all other behaviours (LPA, MVPA)	Multivariable Regression^a^			
	B	95% CI	β	*p* value
Age (years)	0.06	0.03, 0.09	0.40	**< 0.001**
Female gender	0.13	0.01, 0.25	0.19	**0.042**
BMI	0.01	0.00, 0.03	0.19	**0.049**
Socioeconomic deprivation (lowest/highest vs. medium low to high)	0.08	−0.04, 0.20	0.12	0.190
Disease Duration	−0.00	−0.02, 0.01	−0.03	0.752
DMARD therapy	0.03	−0.09, 0.16	0.05	0.590
Comorbidity	−0.03	−0.14, 0.09	−0.04	0.668

^a^Multivariable linear regression model adjusted for period of data collection (April to October vs. November to March).

B, unstandardized regression coefficient; *CI,* confidence interval; *β,* standardized regression coefficient;* LPA,* light intensity physical activity; *MVPA,* moderate-to-vigorous intensity physical activity; *BMI,* body mass index; * DMARD,* Disease modifying antirheumatic drug. Level of significance: *p* < 0.05. Significant results are highlighted in bold

### Movement behaviour composition in JIA patients compared to population controls

As shown in Table 4, patients and matched controls did not differ significantly in terms of age, sex distribution, BMI, height and weight, socioeconomic deprivation, and period of data collection.

**Table 4:** Physical activity and sedentary behavior patterns in JIA patients and controls

Variables	JIA (*n* = 126)	controls (*n* = 126)	*p*-value
Sociodemographic/anthropometric data			
Age, years, mean ± SD	15.0 ± 2.1		-
Female, no. (%)	84 (66.7)		-
BMI (kg/m^2^), mean ± SD	21.1 ± 4.1	21.7 ± 3.6	0.136
Height, cm, mean ± SD	165.7 ± 9.8	166.8 ± 10.4	0.218
Weight, kg, mean ± SD	58.5 ± 15.0	60.7 ± 13.0	0.101
Socioeconomic deprivation*, no. (%)			0.558
Lowest (quintile 1)	22 (18.6)	26 (20.6)	-
Medium-low to medium-high (quintile 2–4)	79 (66.9)	81 (64.3)	-
Highest (quintile 5)	17 (14.4)	19 (15.1)	-
**Accelerometer-specific data**			
Period of data collection, mean (%)			0.544
April to October	57 (45.2)	51 (40.5)	-
November to March	69 (54.8)	75 (59.5)	-
Valid wear days^¥^, no. ± SD	6.6 ± 0.6	6.7 ± 0.6	0.140
Accelerometer wear time, min day^− 1^ ± SD	861.1 ± 118.8	860.7 ± 115.1	0.835
Sedentary time, min day^− 1^ ± SD	740.6 ± 122.9	669.9 ± 114.0	**< 0.001**
Light PA, min day^− 1^ ± SD	71.4 ± 24.5	148.2 ± 44.0	**< 0.001**
Moderate PA, min day^− 1^ ± SD	14.6 ± 6.4	13.9 ± 6.3	0.234
Vigorous PA, min day^− 1^ ± SD	34.5 ± 16.0	24.3 ± 12.1	**< 0.001**
Moderate-to-vigorous PA, min day^− 1^ ± SD	49.1 ± 19.1	38.2 ± 16.1	**< 0.001**
Sedentary time, % day^− 1^ ± SD	85.8 ± 4.7	77.7 ± 6.7	**< 0.001**
Light PA, % day^− 1^ ± SD	8.4 ± 3.1	17.3 ± 4.9	**< 0.001**
Moderate PA, % day^− 1^ ± SD	1.7 ± 0.8	1.6 ± 0.7	0.230
Vigorous PA, % day^− 1^ ± SD	4.1 ± 2.0	2.8 ± 1.4	**< 0.001**
Moderate-to-vigorous PA, % day^− 1^ ± SD	5.8 ± 2.4	4.5 ± 1.9	**< 0.001**
Adherence to WHO PA recommendations, no. (%)	30 (23.8)	10 (7.9)	**0.002**
Adherence to national PA recommendations, no. (%)	4 (3.2)	1 (0.8)	0.375
**Weekdays**			
Valid wear days^¥^, mean ± SD	4.7 ± 0.5	4.9 ± 0.4	**0.024**
Accelerometer wear time, min day^− 1^ ± SD	893.4 ± 117.3	892.5 ± 118.5	0.904
Sedentary time, % day^− 1^ ± SD	85.8 ± 4.8	77.6 ± 6.8	**< 0.001**
Light PA, % day^− 1^ ± SD	8.3 ± 3.1	17.1 ± 4.9	**< 0.001**
Moderate PA, % day^− 1^ ± SD	1.7 ± 0.8	1.7 ± 0.7	0.392
Vigorous PA, % day^− 1^ ± SD	4.2 ± 2.1	3.0 ± 1.5	**< 0.001**
Moderate-to-vigorous PA, % day^− 1^ ± SD	5.9 ± 2.5	4.7 ± 2.0	**< 0.001**
**Weekend days**			
Valid wear days^¥^, mean ± SD	1.8 ± 0.4	1.8 ± 0.4	0.724
Accelerometer wear time, min day^− 1^ ± SD	773.8 ± 156.8	775.8 ± 144.7	0.719
Sedentary time, % day^− 1^ ± SD	85.7 ± 6.8	77.7 ± 10.4	**< 0.001**
Light PA, % day^− 1^ ± SD	8.8 ± 4.1	17.9 ± 6.8	**< 0.001**
Moderate PA, % day^− 1^ ± SD	1.6 ± 1.1	1.5 ± 1.0	0.113
Vigorous PA, % day^− 1^ ± SD	3.9 ± 2.9	2.3 ± 2.2	**< 0.001**
Moderate-to-vigorous PA, % day^− 1^ ± SD	5.5 ± 3.5	3.7 ± 2.9	**< 0.001**

Data are presented as mean ± standard deviation for continuous variables and as absolute and relative frequencies for categorical variables. Presented p-values for comparisons between patients and controls are based on Wilcoxon signed-rank tests for continuous and McNemar tests for categorical variables. JIA, juvenile idiopathic arthritis; BMI, body mass index; PA, physical activity; WHO, World Health Organization. *based on quintiles of the German Index of Socioeconomic Deprivation (GISD)[37]. ^¥^wear time of at least 8 h/day. % day^− 1^. Level of significance: *p* < 0.05. Significant results are highlighted in bold.

Very similar to the patient group, general population controls wore their accelerometer for an average of 6.7 days (SD 0.6) for 14.3 h each.

**Fig. 1:**
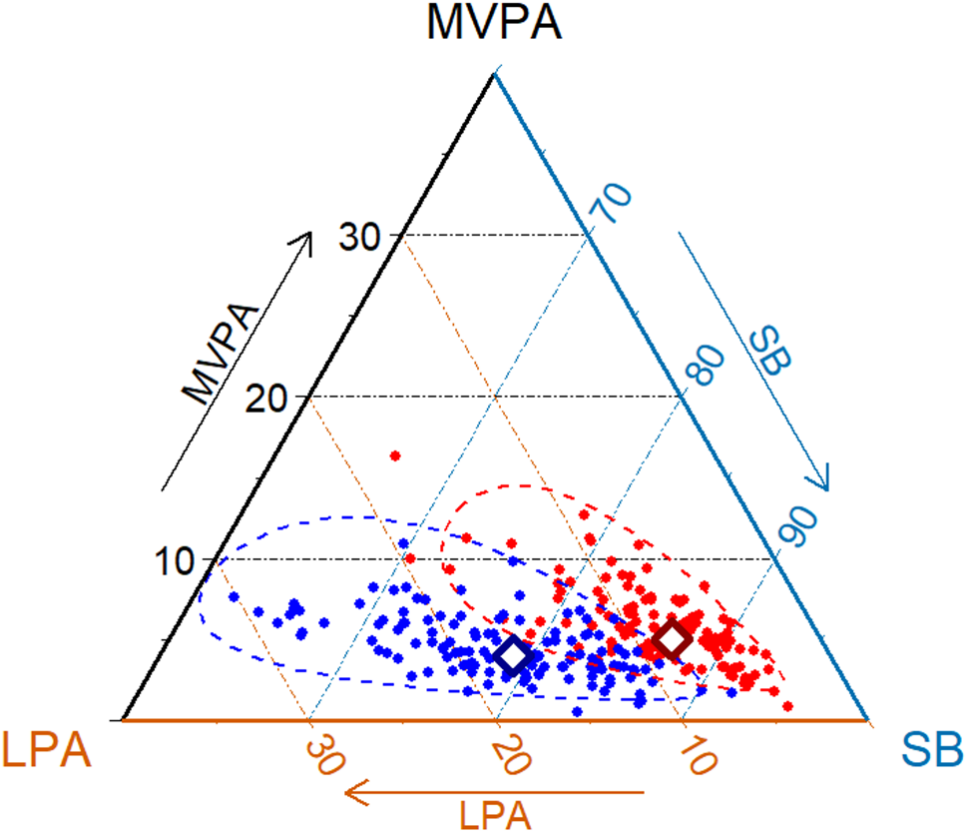
Ternary plot of the samples’ movement behaviour composition Legend: *MVPA,* moderate-to-vigorous physical activity; *LPA,* light-intensity physical activity; *SB,* sedentary behaviour (all in % of 100% accelerometer wear time). With means (white diamonds) and 95% confidence intervals (dashed lines), separately for patients with JIA (red) and matched population controls (blue)

Relative to wear time, patients spent more time in SB and vigorous PA and less time in light-intensity PA, while no significant difference was observed in moderate PA (MPA) compared to controls (all *p* < 0.001, except MPA) (Table 4). These differences were also reflected in the compositional means, illustrating distinct movement behaviour compositions between the groups (Fig. 1). Group differences remained statistically significant in multivariate analysis adjusting for BMI, socioeconomic deprivation, and season of data collection (Wilks’ Λ = 0.31, F(3, 236) = 176.65, *p* < 0.001). The estimated effect size using partial eta squared (η^2^) was 0.69. Comparable patterns were observed in weekday and weekend analyses.

As observed in the patient group, time-use composition of movement behaviours in controls did not differ between weekdays and weekend days.

Almost 24% of patients and 8% of controls achieved the WHO recommended minimum amount of MVPA, while 3% of patients and almost 1% of controls met the national PA guidelines of an average of 90 min MVPA per day (Table 4). Among the controls meeting WHO criteria, 20% were still classified as predominantly sedentary, compared to 87% in the patient group. Notably, these proportions were similar in patients and controls across all age groups. However, both groups showed differences in movement behaviour composition with age, characterized by increasing SB and decreasing light-intensity PA (Table S2), regardless of gender. In both patients and controls, males achieved the WHO recommendation on PA proportionally more often than females (Table S3).

## Discussion

This first multicenter study using device-based data and a compositional analytical approach provides novel insights into daily movement behaviour in young people with well-controlled JIA, compared to controls. Despite low disease activity, most patients exhibited predominantly sedentary routines, with consistently low engagement in light-intensity PA. These patterns remained largely stable across age, gender, and day type. This suggests that behavioural limitations may persist even when overt clinical symptoms are absent.

Moreover, although almost one in four patients met the WHO-recommended MVPA threshold [6], almost all of them were still classified as sedentary based on their overall time-use composition. This paradox illustrates a major limitation of threshold-based classifications. It reinforces the need to consider the full distribution of movement behaviours throughout a day. This is particularly relevant for adolescents with JIA, whose ability to engage in PA may fluctuate due to symptoms such as fatigue or pain.

SB increased with age, primarily at the expense of light-intensity PA, and was more pronounced in females than in males. Meeting MVPA guidelines was more common among male patients, those with oligoarthritis, and individuals from regions of medium socioeconomic deprivation than among their respective counterparts.

In a compositional regression model adjusted for potential confounders, higher relative time spent in SB was significantly associated with female gender, increasing age, and higher BMI. Compared to matched controls, JIA patients spent relatively more time in SB and MVPA, while engaging less in light-intensity PA. These differences remained statistically significant after adjustment and were consistent across age groups and genders. Given the growing consensus that “every minute counts” and that the composition of daily movement is an important predictor of health [6, 42], these less favourable patterns may have clinical relevance.

As expected, and consistent with previous findings in oligo- and polyarticular JIA [16], our patients spent most of their day sedentary. However, the sedentary time observed in our study was higher than that reported in a previous Norwegian single-center study [16]. This difference may be explained by demographic, clinical, or methodological variations between the studies. Possible contributing factors include differences in JIA categories, socioeconomic background, or device parameters such as epoch length and cut-points. In addition, while sensitivity analyses and patient self-reports did not indicate systematic pandemic-related effects within our cohort, contextual factors related to the post-pandemic adjustment period may still partly explain subtle differences in activity routines between studies.

By using validated, age-appropriate thresholds and following ICAD-compliant protocols, our study provides a strong and transparent basis for future research in this field. Importantly, many previous studies did not report SB, did not use age-specific thresholds, or included small and less diverse samples in terms of gender and JIA categories [14–19]. In contrast, our sample reflects the expected distribution of JIA in Germany [33, 34], improving generalizability to clinical care settings.

The observed dominance of sedentary time is concerning, especially in light of evidence linking prolonged SB to low-grade inflammation, independent of PA or adiposity [43]. To our knowledge, this is the first JIA study to demonstrate that relative sedentary time increases with age and is more pronounced in females, findings that remained robust after adjustment. These results are consistent with recent self-reported data from a large German JIA cohort [44] and national paediatric data [45].

The decline in PA with age appears multifactorial and has similarly been observed in non-JIA youth samples. Common explanations include reduced access to suitable facilities, time constraints, increasing autonomy, or diminished social support from family and peers [46].

Like previous studies, we found no association between SB and disease duration [16]. However, we could not replicate the previously reported link between SB and DMARD therapy. Variations in treatment regimens, disease spectrum, or healthcare access may contribute to this discrepancy.

Our study adds to the growing body of literature by applying CoDA. This approach explicitly models the interdependent structure of time-use data and avoids common pitfalls such as collinearity and closure [9–11]. In paediatric rheumatology, this approach enables more accurate estimation of associations and supports nuanced interpretation of movement patterns.

Most previous device-based JIA studies, except for one [16], focused narrowly on MVPA, with little attention to SB or light PA [14, 15, 17–19, 47]. This may be due to the way WHO guidelines prioritize MVPA [6]. However, as our data show, almost all patients who met MVPA guidelines would still be classified as sedentary based on their overall time-use composition, using definitions by the SBRN [31]. In contrast, this pattern was much less frequent among controls, highlighting the particularly unbalanced movement profiles in JIA. These findings further underscore the importance of assessing the full spectrum of movement behaviours in this population, especially given that fluctuating symptoms (e.g., pain) may impede sustained PA.

In our sample, most of the day was spent in SB and light-intensity PA, reinforcing the relevance of promoting lower-intensity activities. These activities may be particularly feasible for adolescents experiencing fluctuating symptoms, offering a practical entry point for behaviour change by utilizing ecological momentary or just-in-time adaptive interventions, based on situational symptom severity. Recent studies have shown that large language models can support the generation of such personalized, context-sensitive prompts in real time, potentially enhancing the effectiveness of digital PA interventions [48].

Around 75% of patients were classified as physically inactive according to the updated WHO guidelines [6]. To our knowledge, this is the first study to apply the revised criteria to JIA. The lower proportion of physically active patients compared to a previous study [18] may reflect the use of stricter, validated cut-points and the higher mean age of participants in our cohort.

Our analysis of weekday vs. weekend behaviour adds a further dimension: movement patterns were stable across both, in line with general population data [47], but contrasting with smaller studies suggesting more MVPA during weekdays [49]. Even if activity composition stays constant, contextual factors such as flexibility and autonomy likely differ. Thus, weekends, typically free of medical appointments, could provide opportunities for interventions promoting light-intensity PA outside structured settings, although these face barriers to implementation [50]. In this context, patient organizations may play a valuable role in supporting weekend activity engagement through peer-based initiatives or local programmes.

We also found that patients with JIA had significantly less favourable movement behaviour compositions than controls. This was primarily driven by higher SB and lower light PA and remained significant after adjusting for confounders. These findings are in contrast to a prior study [16] but supported by another [12].

Given the well-documented adverse outcomes of prolonged SB, such as metabolic and mental health issues [6], our findings further reinforce the need to reduce sedentary time in JIA. This is especially relevant given high rates of mental health problems in adolescents with JIA [3, 51], which often coincide with motivational challenges [52] and may impair engagement in MVPA [53].

One possible approach to addressing these interrelated challenges is regular participation in sports. In the largest mental health screening conducted to date in this population, we found that individuals who reported participating in sports had lower odds of a positive screening result, independent of demographic and clinical factors [51]. Although the type and intensity of activities were not assessed, and causality cannot be inferred, the findings point to potential psychosocial benefits of regular sports participation. However, access to sports may be hindered for some adolescents, especially when low self-efficacy intersects with gender or socioeconomic disadvantage, highlighting the need for inclusive, low-barrier health promotion strategies [54].

Light-intensity activities, such as walking or light chores, require less volitional effort and may provide a feasible entry point for promoting more balanced movement patterns, particularly among adolescents facing fatigue, elevated BMI, or psychosocial barriers.

Interestingly, our JIA cohort accumulated slightly more MVPA than controls, despite a less favourable overall movement behaviour composition. This was mainly due to slightly higher levels of vigorous PA, which contributed disproportionately to their MVPA total. While this contrasts with earlier studies reporting reduced vigorous activity in JIA [14–16], it exceeds the findings of others that observed no significant group differences [19, 47]. Such inconsistencies likely reflect the multifactorial nature of vigorous PA, shaped by factors including age, gender, access to facilities, and individual motivation [55]. However, the small absolute difference in vigorous PA, occurring within a composition otherwise dominated by sedentary behaviour and low light-intensity PA, is unlikely to reflect a clinically meaningful advantage. Given the study’s compositional perspective, which questions the utility of isolated thresholds, this higher level of vigorous PA should not be overinterpreted. While this does not represent sustained, self-initiated behaviour change, such contexts may serve as a valuable entry point for further intervention. Brief physical activity counselling in health care settings has shown moderate success in increasing activity levels, particularly among individuals with chronic conditions [56]. Embedding motivational elements or planning strategies (e.g., implementation intentions) into these contacts could further enhance their impact.

A key strength of this study is the inclusion of a large and well-characterized multicenter cohort of young people with JIA. This was the first study of its kind to cover all ILAR-defined categories and to reflect the expected gender distribution. The device-based assessment of movement behaviour addresses known limitations of self-report measures, particularly in paediatric populations where recall and social desirability bias are common. This study provides a robust and clinically relevant view of daily activity patterns in JIA. It addresses weekday versus weekend differences, uses validated, age-specific intensity thresholds, and includes a broad set of clinical and behavioural outcomes. The use of CoDA further ensures appropriate handling of the co-dependent nature of time-use data. High adherence to wear protocols and ICAD-compliant data processing additionally support the robustness and reproducibility of findings. Together, these methodological decisions offer a strong foundation for future studies aiming to improve the comparability and interpretability of accelerometer-based research in paediatric rheumatology.

It is important to acknowledge several limitations. First, generalizability may be restricted by potential selection bias, as families with higher health awareness or interest in PA might have been more likely to participate. Second, although we excluded the first measurement day to minimize reactivity, subtle behavioural changes may still have occurred. Third, inherent limitations of accelerometers, particularly in detecting activities such as swimming, cycling, or resistance training, may have led to underestimations in certain activity domains; however, non-wear activities such as water-based activities were reported very infrequently and comparable between groups. Despite accounting for age and gender through matching and adjusting for key confounders such as BMI, socioeconomic deprivation, and season, some residual measurement bias may still be present. Furthermore, contextual factors such as peer support, family environment, or weather conditions were not captured. These may have influenced participants’ daily movement behaviours. Finally, the cross-sectional design of the study precludes causal interpretations regarding the observed associations.

In addition, the potential influence of the COVID-19 pandemic should be considered, as recruitment and data collection partly coincided with pandemic periods. However, all assessments took place outside of lockdown phases, during times when schools, recreational, and sports facilities were open. Moreover, patient self-reports and a sensitivity analysis (comparing early vs. late recruits) did not indicate any systematic influence on movement behaviour. While subtle individual-level effects cannot be entirely ruled out, systematic bias due to pandemic-related disruptions appears unlikely.

## Conclusions

This study provides novel insights into the movement behaviour composition of young people with JIA, based on high-quality, device-based data and a compositional analytical approach. Despite well-controlled disease activity, most patients followed predominantly sedentary routines and showed low engagement in light-intensity PA. These patterns were consistent across age, gender, and day type, suggesting that behavioural limitations may persist even in the absence of overt clinical symptoms and may reflect underlying psychosocial impairments.

The finding that many patients met MVPA guidelines yet were still classified as sedentary illustrates the limitations of threshold-based definitions and highlights the value of composition-sensitive assessment. Compared to matched controls, patients demonstrated a significantly less favourable movement behaviour composition. This difference remained significant after adjustment for BMI, socioeconomic deprivation, and season, underlining the clinical relevance of these findings.

Given the marked low level of light-intensity PA and the dominance of SB, light, low-threshold activities may represent a realistic and accessible entry point for improving overall movement balance, particularly in individuals experiencing fatigue, elevated BMI, or psychosocial challenges. By capturing the full context of daily movement behaviours, CoDA offers a robust framework to inform personalised, lifestyle-based recommendations in JIA care. Future research should continue to apply transparent methodologies, include sleep data, and account for contextual factors to support comprehensive 24-hour movement profiling.

## Supplementary Information

Supplementary material 1.

## Data Availability

The datasets used and/or analysed during the current study are available from the corresponding author on reasonable request.

## Abbreviations

ActiMON: Activity monitoring in adolescents and young adults with inflammatory rheumatic musculoskeletal diseases
BMI: Body Mass Index
C-HAQ: Childhood Health Assessment Questionnaire
CoDA: Compositional Data Analysis
bDMARDs: biological disease-modifying antirheumatic drugs
csDMARDs: conventional synthetic disease-modifying antirheumatic drugs
ERA: Enthesitis-related arthritis
GCs: Glucocorticoids
GISD: German Index of Socioeconomic Deprivation
ICAD: International Children's Accelerometry Database
ILAR: International League of Associations for Rheumatology
ILR: Isometric log-ratios
JIA: Juvenile idiopathic arthritis
KiGGS: German Health Interview and Examination Survey for Children and Adolescents
MoMo: Motorik-Modul study
MPA: Moderate physical activity
MVPA: Moderate-to-vigorous intensity physical activity
NPRD: National Paediatric Rheumatological Database
NRS: Numerical Rating Scale
NSAID: Non-steroidal anti-inflammatory drug
PA: Physical Activity
PGA: Physician's global assessment
SB: Sedentary behaviour
SBRN: Sedentary Behaviour Research Network
