# Supplementary material for: Compositional patterns of device-measured movement behaviour in juvenile idiopathic arthritis: results from the multicentre ActiMON study
Source: Arthritis Res Ther. 2025 Dec 17;28:14. doi: 10.1186/s13075-025-03714-5 (PMC12822130; doi:10.1186/s13075-025-03714-5)
Supplement: Supplementary file 1 — Supplementary material 1. [file 13075_2025_3714_MOESM1_ESM.docx]

**Table S1** List of accelerometer data processing criteria (recommended by Migueles et al. [29])

| **Accelerometer data processing criterion** | **Definitions ActiMON** | **Definitions MoMo** |
| --- | --- | --- |
| Accelerometer device | ActiGraph (model: wGT3X-BT) | ✓ |
| Placement of the device | Laterally on top of the right anterior superior iliac spine | ✓ |
| Sampling frequency | 30 Hz | ✓ |
| Filter | Normal ActiGraph GT3X filter | ✓ |
| Epoch length | 1 second | ✓ |
| Non-wear time definition | Choi et al 2011 [55]: 90-minute time window for consecutive zero/nonzero counts; allowance of 2-minute intervals of nonzero counts with an up/downstream 30-minute consecutive zero counts window | ✓ |
| Valid days/valid weeks | 8 hours of recording on at least 4 weekdays and one additional weekend day | ✓ |
| Sedentary and physical activity intensity classification and cut-point algorithms | Romanzini et al 2014 [30] | ✓ |

Same definition = ✓

ActiMON, Activity monitoring in adolescents and young adults with inflammatory rheumatic musculoskeletal diseases. MoMo, Motorik-Modul study.

**Table S2:** Physical Activity and Sedentary Time in JIA patients compared to controls, categorized by age group

| **Variables** | **Age group 10 to 13 years** | | **Age group 14 to 16 years** | | | **Age group 17 to 20 years** | |
| --- | --- | --- | --- | --- | --- | --- | --- |
|  | **JIA** (n=46) | **Controls** (n=46) | **JIA** (n=42) | | **Controls** (n=42) | **JIA** (n=38) | **Controls** (n=38) |
| **Sociodemographic / anthropometric data** |  | |  | | |  | |
| Age, years, mean ± SD | 12.7 ± 0.9 | | 15.2 ± 0.6 | | | 17.5 ± 0.9 | |
| Female, no. (%) | 30 (65.2) | | 27 (64.3) | | | 27 (71.1) | |
|  |  | |  | | |  | |
| **Accelerometer-specific data** |  | |  | | |  | |
| Valid wear days*, no. ± SD | 6.6 ± 0.6 | 6.8 ± 0.6 | 6.6 ± 0.6 | 6.5 ± 0.8 | | 6.5 ± 0.6 | 6.7 ± 0.6 |
| Accelerometer wear time, min day^−1^ ± SD | 838.1 ± 66.1 | 845.4 ± 119.0 | 895.1 ± 171.3 | 882.9 ± 154.8 | | 851.4 ± 88.1 | 853.7 ± 96.7 |
| Sedentary time, % day^−1^ ± SD | 83.6 ± 4.8 | 75.2 ± 7.2 | 86.6 ± 4.5 | 78.5 ± 6.9 | | 87.5 ± 4.1 | 79.8 ± 5.1 |
| Light PA, % day^−1^ ± SD | 10.3 ± 3.0 | 19.4 ± 5.3 | 7.6 ± 2.8 | 16.3 ± 4.4 | | 6.9 ± 2.2 | 15.8 ± 4.2 |
| Moderate PA, % day^−1^ ± SD | 2.0 ± 0.9 | 1.9 ± 0.7 | 1.6 ± 0.7 | 1.5 ± 0.6 | | 1.5 ± 0.6 | 1.5 ± 0.8 |
| Vigorous PA, % day^−1^ ± SD | 4.0 ± 2.0 | 2.8 ± 1.4 | 4.1 ± 1.7 | 2.8 ± 1.5 | | 4.1 ± 2.2 | 2.8 ± 1.4 |
| Moderate-to-vigorous PA, % day^−1^ ± SD | 6.0 ± 2.5 | 4.7 ± 1.8 | 5.7 ± 2.1 | 4.3 ± 1.9 | | 5.6 ± 2.7 | 4.3 ± 1.9 |
| Adherence to WHO PA recommendations, no. (%) | 11 (23.9) | 4 (8.7) | 10 (23.8) | 3 (7.1) | | 9 (23.7) | 3 (7.9) |
| Adherence to national PA recommendations, no. (%) | 2 (4.3) | 0 (0) | 0 (0) | 1 (2.4) | | 2 (5.3) | 0 (0) |
|  |  | |  | | |  | |
| **Weekdays** |  | |  | | |  | |
| Valid wear days*, mean ± SD | 4.7 ± 0.5 | 4.9 ± 0.3 | 4.7 ± 0.5 | 4.8 ± 0.4 | | 4.7 ± 0.4 | 4.9 ± 0.3 |
| Accelerometer wear time, min day^−1^ ± SD | 871.4 ± 70.9 | 882.5 ± 119.9 | 923.6 ± 168.2 | 910.4 ± 139.2 | | 886.7 ± 85.8 | 884.8 ± 89.4 |
| Sedentary time, % day^−1^ ± SD | 83.8 ± 4.9 | 75.2 ± 7.2 | 86.4 ± 4.5 | 78.6 ± 6.9 | | 87.5 ± 4.1 | 79.5 ± 5.3 |
| Light PA, % day^−1^ ± SD | 10.1 ± 3.1 | 19.0 ± 5.1 | 7.5 ± 2.8 | 16.1 ± 4.5 | | 6.8 ± 2.1 | 15.8 ± 4.5 |
| Moderate PA, % day^−1^ ± SD | 2.0 ± 0.8 | 1.9 ± 0.7 | 1.6 ± 0.9 | 1.5 ± 0.6 | | 1.5 ± 0.7 | 1.5 ± 0.8 |
| Vigorous PA, % day^−1^ ± SD | 4.0 ± 2.0 | 3.0 ± 1.5 | 4.4 ± 1.9 | 3.0 ± 1.6 | | 4.2 ± 2.2 | 3.1 ± 1.5 |
| Moderate-to-vigorous PA, % day^−1^ ± SD | 6.0 ± 2.5 | 4.9 ± 2.0 | 6.0 ± 2.4 | 4.5 ± 2.0 | | 5.7 ± 2.7 | 4.6 ± 1.9 |
|  |  | |  | | |  | |
| **Weekend days** |  | |  | | |  | |
| Valid wear days*, mean ± SD | 1.9 ± 0.3 | 1.9 ± 0.3 | 1.8 ± 0.4 | 1.7 ± 0.4 | | 1.8 ± 0.4 | 1.8 ± 0.4 |
| Accelerometer wear time, min day^−1^ ± SD | 751.1 ± 96.7 | 752.7 ± 117.0 | 819.5 ± 209.1 | 805.9 ± 193.7 | | 750.9 ± 140.3 | 770.5 ± 104.1 |
| Sedentary time, % day^−1^ ± SD | 83.2 ± 6.7 | 75.2 ± 9.0 | 86.7 ± 7.2 | 78.0 ± 13.0 | | 87.5 ± 5.7 | 80.4 ± 7.8 |
| Light PA, % day^−1^ ± SD | 10.7 ± 4.0 | 20.7 ± 7.2 | 8.1 ± 4.2 | 16.7 ± 6.2 | | 7.3 ± 3.1 | 15.9 ± 6.0 |
| Moderate PA, % day^−1^ ± SD | 2.0 ± 1.4 | 1.8 ± 1.1 | 1.5 ± 0.9 | 1.4 ± 0.9 | | 1.3 ± 0.9 | 1.2 ± 0.9 |
| Vigorous PA, % day^−1^ ± SD | 4.1 ± 2.7 | 2.3 ± 1.7 | 3.6 ± 3.2 | 2.1 ± 2.1 | | 3.8 ± 2.8 | 2.4 ± 2.7 |
| Moderate-to-vigorous PA, % day^−1^ ± SD | 6.1 ± 3.4 | 4.1 ± 2.6 | 5.1 ± 3.7 | 3.5 ± 2.8 | | 5.1 ± 3.4 | 3.6 ± 3.3 |

PA, physical activity; WHO, World Health Organization. *wear time of at least 8h/day. % per day = relative to accelerometer wearing time.

**Table S3:** Physical Activity and Sedentary Time in JIA patients compared to controls, categorized by gender

| **Variables** | **Females** | | **Males** | |
| --- | --- | --- | --- | --- |
|  | **JIA** (n=84) | **Controls** (n=84) | **JIA** (n=42) | **Controls** (n=42) |
| **Sociodemographic / anthropometric data** |  | |  | |
| Age, years, mean ± SD | 15.1 ± 2.2 | | 14.8 ± 2.0 | |
| BMI, mean ± SD | 20.8 ± 3.8 | 21.8 ± 3.4 | 21.9 ± 4.7 | 21.2 ± 3.8 |
|  |  | |  | |
| **Accelerometer-specific data** |  | |  | |
| Valid wear days*, no. ± SD | 6.7 ± 0.5 | 6.8 ± 0.6 | 6.4 ± 0.7 | 6.5 ± 0.8 |
| Accelerometer wear time, min day^−1^ ± SD | 865.3 ± 105.2 | 865.6 ± 125.6 | 852.8 ± 143.1 | 851.0 ± 91.1 |
| Sedentary time, % day^−1^ ± SD | 86.5 ± 4.3 | 78.3 ± 6.2 | 84.5± 5.4 | 76.6 ± 7.7 |
| Light PA, % day^−1^ ± SD | 7.9 ± 2.8 | 17.0 ± 4.7 | 9.3 ± 3.5 | 17.9 ± 5.4 |
| Moderate PA, % day^−1^ ± SD | 1.7 ± 0.8 | 1.6 ± 0.7 | 1.8 ± 0.8 | 1.7 ± 0.7 |
| Vigorous PA, % day^−1^ ± SD | 3.9 ± 1.8 | 2.7 ± 1.2 | 4.4 ± 2.2 | 3.1 ± 1.8 |
| Moderate-to-vigorous PA, % day^−1^ ± SD | 5.6 ± 2.2 | 4.3 ± 1.6 | 6.2 ± 2.7 | 4.8 ± 2.3 |
| Adherence to WHO PA recommendations, no. (%) | 18 (21.4) | 4 (4.8) | 12 (28.6) | 6 (14.3) |
| Adherence to national PA recommendations, no. (%) | 3 (3.6) | 0 (0) | 1 (2.4) | 1 (2.4) |
|  |  | |  | |
| **Weekdays** |  | |  | |
| Valid wear days*, mean ± SD | 4.8 ± 0.4 | 4.9 ± 0.3 | 4.6 ± 0.6 | 4.8 ± 0.4 |
| Accelerometer wear time, min day^−1^ ± SD | 901.8 ± 106.6 | 899.3 ± 126.7 | 876.7 ± 136.1 | 878.9 ± 100.4 |
| Sedentary time, % day^−1^ ± SD | 86.6 ± 4.1 | 78.2 ± 6.4 | 84.2 ± 5.5 | 76.4 ± 7.5 |
| Light PA, % day^−1^ ± SD | 7.7 ± 2.7 | 16.7 ± 4.7 | 9.4 ± 3.5 | 17.9 ± 5.3 |
| Moderate PA, % day^−1^ ± SD | 1.7 ± 0.7 | 1.6 ± 0.7 | 1.9 ± 1.0 | 1.7 ± 0.8 |
| Vigorous PA, % day^−1^ ± SD | 4.0 ± 1.9 | 2.9 ± 1.3 | 4.5 ± 2.2 | 3.4 ± 1.9 |
| Moderate-to-vigorous PA, % day^−1^ ± SD | 5.7 ± 2.2 | 4.5 ± 1.7 | 6.4 ± 2.9 | 5.1 ± 2.4 |
|  |  | |  | |
| **Weekend days** |  | |  | |
| Valid wear days*, mean ± SD | 1.9 ± 0.3 | 1.9 ± 0.3 | 1.8 ± 0.4 | 1.7 ± 0.5 |
| Accelerometer wear time, min day^−1^ ± SD | 767.7 ± 141.0 | 777.9 ± 156.9 | 786.2 ± 185.7 | 771.4 ± 118.2 |
| Sedentary time, % day^−1^ ± SD | 86.1 ± 6.0 | 78.3 ± 7.8 | 84.7 ± 8.2 | 76.6 ± 14.2 |
| Light PA, % day^−1^ ± SD | 8.5 ± 3.8 | 18.0 ± 6.2 | 9.4 ± 4.6 | 17.7 ± 8.0 |
| Moderate PA, % day^−1^ ± SD | 1.7 ± 1.2 | 1.4 ± 1.0 | 1.5 ± 0.9 | 1.5 ± 1.1 |
| Vigorous PA, % day^−1^ ± SD | 3.6 ± 2.4 | 2.2 ± 2.1 | 4.3 ± 3.7 | 2.4 ± 2.2 |
| Moderate-to-vigorous PA, % day^−1^ ± SD | 5.3 ± 3.1 | 3.6 ± 2.7 | 5.8 ± 4.3 | 3.9 ± 3.1 |

PA, physical activity; WHO, World Health Organization. *wear time of at least 8h/day. % per day = relative to accelerometer wearing time.
